# THE CO-EVOLUTION OF PERSONAL NETWORKS AND LONELINESS FOLLOWING WIDOWHOOD FOR MEN AND WOMEN

**DOI:** 10.1093/geroni/igad104.1841

**Published:** 2023-12-21

**Authors:** Jina Lee, Markus Schafer

**Affiliations:** University of Toronto, Toronto, Ontario, Canada; Baylor University, Waco, Texas, United States

## Abstract

Late-life transitions such as widowhood often re-calibrate people’s close personal networks. Some network change—particularly growth in network size and closer geographic access to network members—is assumed to protect against loneliness, but scholars have yet to systematically examine these processes post widowhood. This study uses data from four waves of the German Aging Survey (DEAS), conducting gender-specific hybrid panel modeling to estimate both within- and between-individual effects of (1) network conditions up to seven years past widowhood, and (2) the effects of network change on loneliness. Results reveal that network size takes on a reversed U-shape: Germans becoming widowed tend to see an influx of new core ties from beyond their family, but this trend slows and reverses with time. There was also some evidence of non-linear change related to distance: geographic distance to core network members tended to shrink following widowhood before expanding back outward. Furthermore, moderation analyses an important role for these network characteristics on loneliness. Larger non-kin networks in the aftermath of widowhood partially alleviated the loneliness associated with that transition. The role of geographic proximity was gender-specific, as widowed men with nearby non-kin ties were most protected against loneliness, whereas widowed women fared best if their kin ties were farther away. Altogether, this study presents novel insight into how personal networks evolve after widowhood, revealing the nuanced, gendered ways that networks adapt. Efforts to reduce loneliness after widowhood may consider how gender roles and expectations shape the transmission and meaning of companionship and support.

